# TGF-β, IL-1β, IL-6 levels and TGF-β/Smad pathway reactivity regulate the link between allergic diseases, cancer risk, and metabolic dysregulations

**DOI:** 10.3389/fimmu.2024.1371753

**Published:** 2024-04-02

**Authors:** Zeev Elkoshi

**Affiliations:** Research and Development Department, Taro Pharmaceutical Industries Ltd, Haifa, Israel

**Keywords:** TGF-β, IL-1β, asthma, allergic rhinitis, cancer risk, obesity, type 2 diabetes, cell metabolism

## Abstract

The risk of cancer is higher in patients with asthma compared to those with allergic rhinitis for many types of cancer, except for certain cancers where a contrasting pattern is observed. This study offers a potential explanation for these observations, proposing that the premalignant levels of circulating transforming growth factor-β (TGF-β), IL-1β, and IL-6 as well as the reactivity of the TGF-β/Smad signaling pathway at the specific cancer site, are crucial factors contributing to the observed disparities. Circulating TGF-β, IL- β and IL-6 levels also help clarify why asthma is positively associated with obesity, Type 2 diabetes, hypertension, and insulin resistance, whereas allergic rhinitis is negatively linked to these conditions. Furthermore, TGF-β/Smad pathway reactivity explains the dual impact of obesity, increasing the risk of certain types of cancer while offering protection against other types of cancer. It is suggested that the association of asthma with cancer and metabolic dysregulations is primarily linked to the subtype of neutrophilic asthma. A binary classification of TGF-β activity as either high (in the presence of IL-1β and IL-6) or low (in the presence or absence of IL-1β and IL-6) is proposed to differentiate between allergy patients prone to cancer and metabolic dysregulations and those less prone. Glycolysis and oxidative phosphorylation, the two major metabolic pathways utilized by cells for energy exploitation, potentially underlie this dichotomous classification by reprogramming metabolic pathways in immune cells.

## Introduction

Asthma and allergic rhinitis (AR) are two allergic conditions classified as Type I Hypersensitivity which is characterized by high IgE antibody release from plasma cells. Allergens are the triggers of allergic reactions. Antigen-presenting cells process allergens and present them to naïve CD4+T cells. In the presence of IL-4, naïve CD4+T cells differentiate into allergen-specific Th2 cells. Simultaneously, B cells process allergens and present them to these allergen-specific Th2 cells through MHC-II. Consequently, these Th2 cells secrete IL-4 and IL-13, initiating the production of antigen-specific IgE antibodies by B cells, a process which leads to the differentiation of B cells into plasma cells that generate substantial amounts of specific IgE antibodies. These IgEs subsequently bind to high-affinity IgE receptors found on the surfaces of mast cells and basophils. Upon re-exposure to the antigen, it can bind to the IgE antibodies already attached to mast cells and basophils, triggering the release of allergy mediators such as histamine, tryptase, proteoglycans, and chymase (1).

Two epidemiological studies examined the risk of cancer in the Swedish population among individuals with asthma or AR. In one study, standardized incidence ratios (SIRs) were assessed for various types of cancer occurrences in a group of 138,723 individuals diagnosed with hay fever or AR. This cohort was identified from three different Swedish healthcare databases. Data from the Swedish Cancer Registry was utilized for the analysis of cancer risk (2). Another study followed a similar methodology, evaluating SIRs for different cancer types in a group of 439,738 Swedish patients with asthma (3). Remarkably, in 16 out of 23 cancer types evaluated:


(1) SIR (asthma patients) > SIR (allergic rhinitis patients)

The overall risk of cancer in asthmatic patients is 1.19 (95% CI: 1.17-1.21) versus 1.03(95% CI: 0.99 -1.07) in allergic rhinitis patients. The data is presented in **Table 1**.

**Table 1:** SIRs for specific cancer types in asthmatic patients and in allergic rhinitis patients.

Cancer Type	SIR (95% C.I.) in asthmatic patients (3)	SIR (95% C.I) in allergic rhinitis patients (2)
Upper aerodigestive tract	1.11 (0.96 - 1.28)	0.84 (0.60 - 1.15)
Esophagus	1.69 (1.40 - 2.01)	0.50 (0.21 - 0.99)
Stomach	1.37 (1.22 - 1.54)	1.06 (0.77 - 1.42)
Colon	1.30 (1.22 -1.39)	0.87 (0.73 - 1.03)
Rectum	1.13 (1.02 - 1.24)	0.89 (0.70 - 1.12)
Liver	1.29 (1.14 - 1.46)	0.62 (0.40 - 0.92)
Pancreas	1.35 (1.19 - 1.52)	0.76 (0.52 - 1.07)
Nose	NA	2.63 (1.19 - 5.01)
Lung	1.97 (1.87 - 2.09)	0.78 (0.64 - 0.93)
Cervix	1.07 (0.88 - 1.30)	0.99 (0.73 - 1.32)
Kidney	1.35 (1.19 - 1.52)	1.31 (1.02 - 1.67)
Urinary bladder	1.26 (1.15 - 1.37)	0.79 (0.62 - 1.00)
Nervous system	1.24 (1.11 - 1.38)	1.15 (0.96 - 1.37)
Thyroid	1.37 (1.10 - 1.69)	1.17 (0.82 - 1.61)
Non-Hodgkin lymphoma	1.04 (0.93 - 1.16)	1.00 (0.82 - 1.22)
Myeloma	1.14 (0.96 - 1.36)	1.08 (0.72 - 1.55)
Leukemia	1.17 (1.05 - 1.30)	0.82 (0.60 - 1.09)
Breast	1.01 (0.95 - 1.06)	1.11 (1.01 - 1.21)
Endometrium	0.74 (0.65 - 0.85)	0.85 (0.65 - 1.09)
Ovary	0.89 (0.76 - 1.04)	0.98 (0.74 - 1.27)
Prostate	1.13 (1.08 - 1.19)	1.18 (1.06 - 1.30)
Testis	1.36 (1.06 - 1.72)	1.46 (1.10 - 1.89)
Melanoma	0.78 (0.70 - 0.88)	1.10 (0.93 - 1.28)
Skin, Squamous cell	1.18 (1.09 -1.28)	1.21 (1.00 - 1.46)

NA, not available.

Endocrine glands related cancer, which appears in the studies by Hemminki and Liu, was omitted in **Table 1**, since it includes pancreatic, prostate, and breast cancers which are separately referred to in the list.

Metabolic dysregulations refer to disruptions or abnormalities in the normal metabolic processes of the body, including energy production, nutrient utilization, and waste elimination. A dysfunction in these metabolic processes can lead to various health issues such as obesity, Type 2 diabetes (DM2), insulin resistance, glucose intolerance dyslipidemia, and hypertension (4). Several studies investigated the association between metabolic dysregulations and allergic diseases. A positive association was found between asthma and DM2 in a recent literature review (5), and between asthma and hypertension (6). A positive association was also found between abdominal obesity and asthma in a meta-analysis published in 2019 (7). The evidence for these findings is substantiated by experimental models. Various murine models of obesity, including ob/ob, db/db, and Cpefat mice, manifest inherent airway hyperresponsiveness, a prominent characteristic of asthma (8). Additionally, other obese mouse models, such as TALLYHO/Jng mice, and mice subjected to a high-fat diet, exhibit asthma-like symptoms upon allergen sensitization and challenge (9). It is important to highlight that ob/ob and db/db mice experience insulin resistance and a gradual deterioration in glycemic control starting from 4 weeks of age (10). Consequently, these mouse models illustrate a direct association between DM2 and asthma. In support of this correlation, a study showed that metformin, an anti-glycemic drug, decreased both serum insulin levels and airway hyperresponsiveness in 12-week-old C57BL/6J mice that had been fed a high-fat diet for 8 weeks (11). In contrast to the positive association observed between metabolic disorders and asthma, a study by Lee et al. (12) reported an inverse association between AR and diabetes mellitus (the study data acquisition was biased towards DM2). Another study reported a lower prevalence of AR in patients with higher levels of fasting plasma glucose (13). A large population-based study in 30,590 Korean patients found that AR prevalence was significantly lower in subjects with metabolic syndrome (OR 0.84; 95% CI 0.76–0.93), high blood pressure (OR 0.85; 95% CI 0.77–0.94), or impaired fasting glucose (OR 0.81; 95% CI 0.73–0.89) (14). Likewise, a reverse association was observed between central obesity in children and AR (15).

In the next sections, a potential explanation for these observations will be proposed.

## The impact of TGF-β on cancer risk in patients with neutrophilic asthma or allergic rhinitis

A notable distinction in the pathophysiology of asthma and allergic rhinitis becomes evident when comparing the circulating levels of TGF-β in both conditions. In one study, mean serum TGF-β levels evaluated in asthmatic patients were *two orders of magnitude* higher than the values observed in normal controls (39.59 ng/ml vs. 0.26 ng/ml). In steroid naϊve mild asthma patients TGF-β levels were even higher (47.44 ng/ml) (16). In another study, the mean value over 70 asthmatic patients (who did not use antihistaminic, oral, or parenteral steroid within the previous 4 weeks before blood sampling) was 41.7 ng/ml compared to a mean of 27.7 ng/ml over 15 healthy controls (*p* < 0.05) (17). Seven-fold increase in mean TGF-β serum levels was reported in 15 patients with moderate asthma (who did not use corticosteroids) compared to 14 healthy controls (36.78 ng/ml vs. 5.49 ng/ml) (18). In another study, TGF-β levels in the serum and bronchoalveolar lavage fluid (BALF) of asthmatic patients were reported as significantly higher compared to controls (serum: 0.186 ng/ml vs 0.119 ng/ml; BALF: 0.215 ng/ml vs 0.121 ng/ml) (for both: *p* < 0.05) (19). On the other hand, a study by Rosas et al. (20) reports significantly lower mean plasma values of TGF-β in AR patients compared to values observed in a control group (0.276 ng/ml vs. 0.932 ng/ml) (*p* < 0.005). Chai et al. (21) also reported a lower mean value for TGF-β blood level (following mitogen stimulation) in AR patients compared to healthy controls (19.1 ng/ml vs. 23.8 ng/ml). In unstimulated blood samples, the mean values were 17.9 ng/ml for AR compared to 23.8 ng/ml in healthy controls. While these differences were not statistically significant, a tendency toward lower TGF-β levels in AR is observed (21). These last results are confounded by the co-existence of asthma in some patients, which likely induces an increase in TGF-β levels. Additionally, some patients received anti-allergic treatment, which is also known to induce an increase in TGF-β levels (22). It is reasonable to assume that the levels of TGF-β in AR would have been lower without these confounding factors. Nevertheless, the values reported by Chai et al. in AR patients are comparable to those reported by Ciprandi et al. (23) in AR patients outside the pollen season. In addition, Qiu et al. (24) demonstrated statistically significant lower TGF-β serum levels in AR patients compared to healthy controls. Another study that evaluated TGFβ mRNA levels in nasal mucosa of AR patients found a statistically significant lower value in AR patents compared to healthy controls (25). It must be noted that TGF-β levels are sensitive to allergen exposure (23, 24), and the levels following exposure to the allergen or allergen-specific immunotherapy are higher compared to the values observed in patients not exposed to the allergen (23, 24). However, even after an exposure to the allergens, TGF-β levels are not higher in AR than those observed in healthy subjects (24).

Despite variations in reported serum TGF-β values among different studies, a consistent observation is that circulating TGF-β levels are significantly higher in individuals with asthma compared to healthy controls. Conversely, in patients with AR who have not been exposed to allergens, TGF-β levels are lower compared to controls. An outstanding result was reported by Bayrak Degirmenci et al. (26) who found that TGFβ levels in AR patients were higher compared to healthy controls. However, the patients in this study were evaluated in the symptomatic period (i.e. after exposure to the allergen).

The asthma patient population comprises several phenotypes. Broadly, asthma may be divided into two main classes: “type 2-high” and “type 2-low” classes. In “type 2-high” asthma, which encompasses about half of the patients, Th2 cytokines, namely IL-4, IL-5, and IL-13, are the primary drivers of the disease, and patients are characterized by high frequencies of allergen-specific immunoglobulin E (sIgE) antibodies in blood. In “type 2-low” asthma, airway inflammation is mediated by IL-1β, IL-6, and neutrophils, with a lack of Th2 response and low frequencies of blood sIgE antibodies (27, 28). This type of asthma, also termed “non-eosinophilic asthma” or “neutrophilic asthma,” involves IL-6 produced by airway epithelial cells and neutrophils, IL-1β produced following inflammasome activation, and TGF-β produced by neutrophils and eosinophils. The ORMDL3 and GSDMB genes are strongly associated with asthma. Elevated levels of ORMDL3 expression in RAW264.7 macrophages result in increased production of IL-6, IL-1β, and TGF-β1. Additionally, it has been observed that GSDMB induces TGF-β1 expression in primary human bronchial epithelial cells (29). It is of note Serum mean IL-23 levels were reported to be significantly higher in untreated severe asthmatics than in a control group of healthy volunteers (111.38 pg/ml vs. 30.63 pg/ml; p< 0.001) (30). In the presence of IL-21 and IL-6, TGF-β induces the differentiation of Th17 cells from naïve CD4+ T cells in *mice* (31), fostering a pro-inflammatory environment. The differentiation of naive *human* CD4+ T cells into Th17 cells requires TGF-β, IL-1β, and either IL-6, IL-21, or IL-23 (32). Neutrophilic asthma is therefore associated with Th17 differentiation. Th17 cells release IL-17, IL-21, and IL-22, which are dominant cytokines in neutrophilic asthma. It has also been reported that the association of asthma with 17q21 polymorphisms in ORMDL3 and GSDMA is linked to early-life secretion of IL-17 (33). IL-17 is responsible for the recruitment of neutrophils into the lungs (34) while neutrophils further contribute to the pro-inflammatory characteristics of the environment by releasing TGF-β and inducing the cleavage of pro-IL-1β to IL-1β (35) (a positive-feedback mechanism). During infection, neutrophils polarize macrophages toward the pro-inflammatory M1 phenotype (36). Similarly, M1 polarization is associated with airway neutrophilia in asthma (37). The rate of Th17 cell differentiation increases with TGF-β levels (up to a point where TGF-β blocks the IL-23 receptor) (31). Therefore, high TGF-β levels (below the turndown point) observed in neutrophilic asthma accelerate Th17 cell differentiation. In addition, the phenotype of neutrophilic patients is characterized by significantly elevated sputum IL-1β gene expression and sputum IL-1β protein levels compared to eosinophilic phenotype (38). Elevated IL-1β levels must also expedite Th17 differentiation.

In summary, high TGF-β levels in asthma compared to AR, and high IL-1β levels in neutrophilic asthma compared to eosinophilic asthma promote a Th17 related pro-inflammatory environment in asthmatic patients especially in patients with neutrophilia.

Along the course of tumor development, the tumor microenvironment (TME) develops from basically anti-tumor at early tumor stage to a pro-tumor environment, at late tumor stages. Neutrophils transform from the pro-inflammatory N1 phenotype to the anti-inflammatory N2 phenotype. Similarly, macrophages transform from the M1 pro-inflammatory phenotype to the M2 anti-inflammatory phenotype. A correlation between regulatory T cells (Treg cells) frequency (in both the TME and circulation) and cancer progression was observed in several types of cancer [ref. (39) and references therein]. Tregs suppress the pro-inflammatory activities of macrophages, neutrophils, NK cells, T cells and B cells (40). In addition, myeloid-derived suppressor cells (MDSC), defined as pathologically activated neutrophils and monocytes with potent immunosuppressive activity (41), accumulate in the circulation and in the TME during cancer progression (42). TGF-β contributes to an immunosuppressive TME. Studies have shown that TGF-β can induce the transition of N1 to N2 (43) and M1 to M2 (44) phenotypes. In the presence of IL-2, TGF-β promotes the differentiation of naïve T cells into suppressive inducible regulatory T cells (iTregs) (45, 46). Moreover, TGF-β enhances the immunosuppressive function of MDSC (47).

Drawing from these observations, the present report proposes that the difference in circulating TGF-β, could be accountable for the increased risk of various cancer types among individuals with *neutrophilic* asthma in comparison to those with AR, while the interference of the TGF-β/Smad signaling pathway leads to an opposite outcome. There are two mechanisms that could contribute to an increased incidence of cancer when TGF-β levels are elevated:

(a) Elevated TGF-β levels *in the context of neutrophilic asthma* create a pro-inflammatory environment that contributes to the initiation and early development of cancer. This effect parallels the impact of pre-existing autoimmune diseases on cancer risk (39). Inflammation has the potential to induce mutations that upregulate oncogenes or mutations that downregulate tumor-suppressor genes, thereby enhancing tumor initiation. Additionally, inflammation fosters cancer cell proliferation, as exemplified by the inactivation of nuclear factor-kappa B (NFκB) in immune cells (48).
(b) TGF-β is implicated in inducing and promoting the self-renewal of cancer stem cells. Cancer stem cell renewal or expansion was reported in glioma (49, 50), triple-negative breast cancer (51), and mammary cancer colonies induced in bone microenvironment (52). In pancreatic cancer, TGF-β enhanced the invasiveness of cancer stem cells (53).

The next two sections present lines of evidence to support the proposed hypothesis.

### When TGF-β signaling is impaired in premalignant cancer tissue, the risk of this specific cancer in asthma patients is not higher than its risk in allergic rhinitis patients

(a) 5α-dihydroxytestosterone (DHT) has been reported to regulate TGF-β signaling in prostate epithelial cells by transcriptionally suppressing TGF-β receptor II (TGF-βRII) (54). Notably, Equation 1 does not apply to prostate cancer (**Table 1**). It does not hold for testicular cancer as well (**Table 1**), as the testicles are a significant site of androgen synthesis.
(b) Estrogens and progesterone have been reported to inhibit TGF-β signaling by reducing the phosphorylation of Smad2/3 (55–57). Consequently, Equation 1 is not applicable to ovarian and endometrial cancers (**Table 1**). Ovarian tissue estrogen levels are at least 100-fold higher than circulating levels (58). Many of the endometrial cancer risk factors involve excess estrogens or estrogen signaling, and endometrial cancer cases usually express high levels of estrogen receptor α (59).
(c) Similar to the sex organs, the skin locally synthesizes considerable amounts of sex hormones (60, 61). No wonder that melanoma and squamous cell skin carcinoma do not obey Equation 1 (**Table 1**).
(d) Benign breast disease is associated with increased estrogen levels in breast tissue (62) and in the circulation (63). Nearly 30% of all breast cancers develop in women with prior benign breast disease (64). This explains why Equation 1 is not obeyed for breast cancer (**Table 1**).

### The high risk of nasal cancer in allergic rhinitis correlates with locally high TGF-β activity and increased IL-1β and IL-6 levels

Nasal cancer shows the highest cancer risk among all cancer types observed in patients with a history of AR (**Table 1**). Consistent with this finding, significantly increased immunoreactivity for TGF-β and TGF-β receptors was reported in the nasal mucosa of AR patients compared to the nasal mucosa of normal controls (65). Increased IL-1β and IL-6 levels (needed for TH17 differentiation) in nasal lavage was reported in AR patients following allergen challenge (66).

## TGF-β levels are linked to the interrelations between neutrophilic asthma or allergic rhinitis, and metabolic dysregulations

Yadav et al. (67) have illustrated the importance of the TGF-β/Smad3 signaling pathway in regulating glucose and energy homeostasis. These authors demonstrated that Smad3 deficient mice are protected from diet-induced obesity and diabetes. Moreover, a strong correlation between TGF-β1 levels and adiposity in rodents and humans was reported in this study. It also became evident that TGF-β1, along with other members of the TGF-β superfamily, disrupts adipogenicity. Adipogenicity involves the **“**healthy**”** expansion of adipose tissue through the proliferation of adipose progenitor cells as a means of storing excess energy. In fact, TGF-β promotes the proliferation of preadipocytes while inhibiting their differentiation into adipocytes. Accumulation of dysfunctional adipose tissue in obesity increases risk for cardiometabolic diseases (68).

Pancreas beta (β) cell dysfunction or loss is the common pathological feature in all types of diabetes mellitus. The TGF-β/Smad signaling pathway has been shown to play a central role in the pathophysiology of pancreatic β cells. More specifically, deletion of Smad3 simultaneously improves pancreatic β cell function, apoptosis, and systemic insulin resistance with the consequence of eliminated overt diabetes in diabetic mouse models (69–71), revealing Smad3 as a key mediator and ideal therapeutic target for DM2. Importantly, IL-6 is constitutively expressed by β and α pancreatic cells from healthy or DM2 patients (72), and IL-1β is produced by α pancreatic cells at comparable levels in both non-diabetic and diabetic (DM1 and DM2) donors (73).

There is evidence of elevated TGF-β production and gene expression by peripheral blood monocytes in hypertensive patients (74, 75). Additionally, hypertensive individuals have been found to exhibit heightened circulating levels of IL-1β and IL-6 (76).

Due to the association of asthma in general, and neutrophilic asthma in particular, with elevated circulating TGF-β levels, the positive connection between neutrophilic asthma and obesity, DM2, and hypertension becomes evident. Similarly, the inverse association between allergic rhinitis, obesity, hypertension and DM2 is also clarified, as allergic rhinitis is linked to reduced circulating TGF-β. It appears that the elevated levels of TGF-β and IL-1β observed in the subset of neutrophilic asthma patients [comprising 20%-30% of asthmatic patients (77)] may be responsible for the differences in the association of asthma and AR with metabolic dysregulations.

It should be noted that elevated levels of IL-1β and IL-6 were reported in AR. Multiple studies, demonstrated an elevation in IL-1β levels in nasal secretion and nasal mucosa of AR patients following allergen exposure (78). An increase in blood levels of IL-1β was reported in children with moderate to severe persistent AR (79). In addition, studies by Mirzai (25) and Gao (80) revealed increased expression of IL-6 in the nasal mucosa and serum, respectively, among patients with AR compared to controls. Nevertheless, despite the elevated levels of IL-1β and IL-6 in AR, the comparatively low levels of TGF-β in AR likely constrain Th17 differentiation.

## Impaired TGF-β/Smad signaling is linked to a decreased risk of cancer in obese individuals

**Figure 1:**
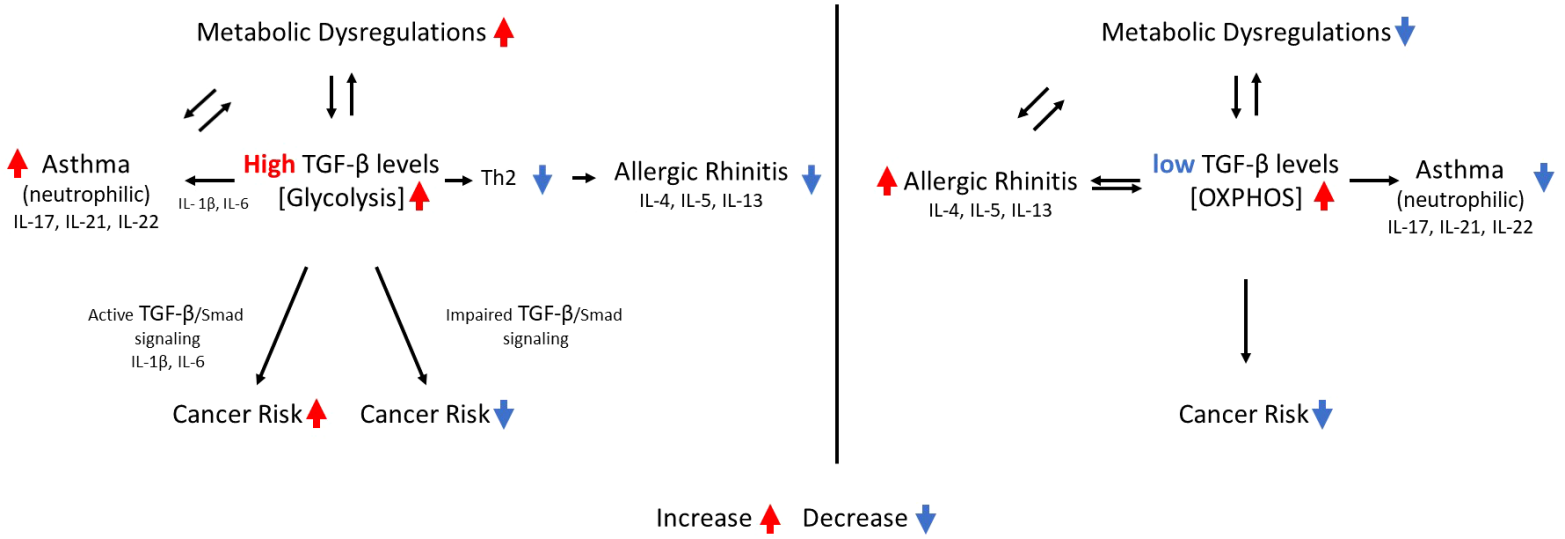
the interrelations between circulating TGF-β levels, asthma, allergic rhinitis, metabolic dysregulations, cancer risk and metabolic pathways. A single arrow indicates a causative effect. Two arrows pointing in opposite directions indicate an association between the two events.

By simple deduction, it turns out that for cancer types with impaired TGF-β signaling (cancer types where Equation 1 is not obeyed), obesity is expected to be associated with a reduced risk of cancer (see **Figure 1**). Five out of seven cancer types with impaired TGF-β signaling indeed demonstrate reduced risk of cancer in obese individuals compared to controls with normal body mass index (BMI): skin melanoma, prostate cancer, breast cancer (premenopausal) (81), skin squamous cell carcinoma (82), and testicular cancer (83, 84). As an exception, BMI is a consistent and leading risk factor for endometrial cancer in premenopausal women (85), another type of cancer where Equation 1 in not obeyed. The link between BMI and ovarian cancer varies among studies (86).

## Discussion

### The strong association between neutrophilic asthma and obesity

A study involving 423 asthma patients found that obese patients demonstrated more neutrophils in sputum and blood than nonobese patients (87). This result is consistent with two other studies (88, 89). In a large study that included 727 adult asthma patients, Todd et al. (90) found no statistically significant difference in sputum neutrophil count among five groups with increasing median BMI. However, in this study, the mean neutrophil count *consistently* increased with increasing BMI. A more recent study reports elevated blood neutrophils with a higher BMI in 166 adolescent asthma patients (91). In another recent study, the serum leptin level was higher in obese than in lean female mice. This study demonstrated that leptin/obR signaling (obR is the leptin receptor), plays an important role in the pathogenesis of obesity-related neutrophilic airway inflammation in females, by promoting M1 macrophage polarization (92). A recent review discusses the contribution of neutrophils to obesity-associated inflammation (93). It is also noted that obesity in non-asthmatic adolescents is associated with increased sputum mRNA expression of IL6, IL8, IL13, IL17, IL23, and IFN-γ (94).

In contrast to neutrophilic asthma, allergic rhinitis inflammation is mainly mediated by Th2 cytokines. Although allergic rhinitis patients demonstrate increased levels of Th17 cytokines as well (95), blood frequencies of Th17 cells in asthmatic children were reported to be statistically significantly higher compared to those in children with allergic rhinitis (96). This difference would likely increase if only children with neutrophilic asthma were included in this study. As a Th2 disease, the presence of IL-4 and IL-13 is needed for AR development. Allergic rhinitis is associated with IL-4 and IL-13 polymorphism (97), and dual blockade of IL-4 and IL-13 with dupilumab, an IL-4Rα antibody, is required to inhibit type 2 inflammation such as AR (98, 99). At the same time, high TGF-β levels which are essential for the development of neutrophilic asthma, inhibits the development of Th2 reactions (100), and hence suppress allergic rhinitis (see **Figure 1**). It must be noted that TGFβ is also produced by eosinophils (as well as epithelial cells, macrophages, and fibroblasts) (101). Yet, the increased levels of IL-1β in neutrophilic asthma (as indicated by Simpson et al. (38)) likely contribute to a more pronounced differentiation of Th17 cells with this phenotype. It was also reported that Th17/Treg homeostasis, but not Th1/Th2 homeostasis, is implicated in the exacerbation of human bronchial asthma (102), underscoring the role of neutrophils in airway inflammation. Collectively, the distinct association of asthma with long-term comorbidities can be primarily attributed to neutrophilic patients, due to the elevated circulatory levels of TGF-β, IL-1β and IL-6 in this type of asthma.

### The binary model of TGF-β reactivity

For simplicity, this study adopts a dichotomous model where high TGF-β activity in conjunction with IL-1β and IL-6 is linked to asthma, metabolic dysregulations, and an elevated risk of cancer, while it is inversely associated with the occurrence of AR. In contrast, low TGF-β activity (with or without the presence of IL-1β and IL-6 levels), are associated with the inverse effects. In previous publications, the binary classification of immune response as **“**high Treg**”** or **“**low Treg**”**, proved useful in elucidating the association of certain pathogens with cancer and the association of others with autoimmune diseases. It also explained why certain specific pathogens are involved in coinfections. The effectiveness or ineffectiveness of specific immune-modulating drugs in the treatment of autoimmunity and cancer was also clarified by this binary model (103). The binary Tregs classification was also found to be useful in predicting the efficacy or inefficacy of several immune-modulating drugs in the treatment of severe coronavirus disease 2019 (COVID-19) (104, 105). Since regulatory T cells are a major source of TGF-β, the **“**high TGF-β**”** or **“**low TGF-β**”** classification is closely related to the **“**high Treg**”** and **“**low Treg**”** classification.

### The binary model of TGF-β reactivity in relation to oxidative phosphorylation and glycolysis

A binary classification of immune responses aligns with the two pathways of nutrients metabolism used by eukaryotic living cells to utilize and store energy: oxidative phosphorylation (OXPHOS) and glycolysis. OXPHOS consists of two closely interconnected components: the electron transport chain, which in eukaryotes occurs in the mitochondria, and chemiosmosis. NADH and succinate, produced in the citric acid cycle, undergo oxidation, releasing energy used by ATP synthase to facilitate the formation of ATP (adenosine triphosphate). The process of glycolysis, on the other hand, occurs in the cytoplasm and produces 2 ATP molecules per glucose molecule. Pyruvate, the end-product of glycolysis, serves as fuel for OXPHOS. Under aerobic conditions, pyruvate enters the mitochondria for oxidation to acetyl CoA, initiating the tricarboxylic acid (TCA) cycle and OXPHOS, which can yield 36 ATPs per glucose molecule. Conversely, under anaerobic conditions, pyruvate is reduced to lactate, by lactate dehydrogenase A (LDH-A) in the cytoplasm. The resulting lactate is then excreted into the extracellular space through monocarboxylate transporters. Normal cells usually release energy for ATP synthesis through OXPHOS (with glucose and fatty acids as fuels). However, most cancer cells preferentially utilize glycolysis, even in the presence of oxygen, a phenomenon known as the Warburg effect (106). While the glycolytic pathway from glucose to pyruvate is less efficient in energy exploitation than OXPHOS, its rate is faster. Inflammatory cells, including Th1, Th17, CD8 T cells, and M1 macrophages, primarily use glycolysis for energy storage. On the other hand, immune cells associated with the anti-inflammatory arm of the immune system, such as Tregs, M2 macrophages, memory T cells, and resting microglia, rely predominantly on OXPHOS and fatty acid oxidation for ATP production (106, 107).

TGF-β has been associated with increased glycolysis in cancers (108–111) and osteoarthritis (112). In addition, as aforementioned, in the absence of IL-1β and/or IL-6, it induces immunosuppression. In fact, Gauthier et al. (113) recently demonstrated that TGF-β uncouples glycolysis and inflammation in macrophages. By enhancing the expression and activity of a glycolytic enzyme, TGF-β induced glycolysis in macrophages, but, at the same time, suppressed their pro-inflammatory cytokines.

Rates of glycolysis in the liver, in pancreas β cells, and in adipose tissue are increased in DM2 due to compensatory hyperinsulinemia (114). It was also reported that aerobic glycolysis is increased in CD4 T cells in asthma (115). Lactate content was significantly higher in sputum supernatants from asthmatic patients, notably those with greater than 61% neutrophils, relative to healthy controls. One source of lactate production in asthmatic patients were primary nasal epithelial cells (116). Taken together, elevated TGF-β levels and increased glycolysis are associated with asthma (especially neutrophilic asthma), with an increased risk of cancer, with obesity, with DM2, hypertension, and with insulin resistance.

No studies describing OXPHOS activity in AR could be found. However, atopic dermatitis, which is associated with a low-producer TGF-β1 cytokine genotype (117), exhibits high OXPHOS activity in nonlesional skin (118). On the other hand, TGF-β1 expression was found to be markedly enhanced in atopic dermatitis lesions (119). In agreement, a mouse model of lesional atopic dermatitis demonstrated metabolic shift toward enhanced anaerobic glycolysis (120). Urticaria shows lower serum TGF-β levels compared to normal levels, but the difference was not statistically significant (121). Mast cells, the primary cells involved in producing urticaria wheals (122), utilize the OXPHOS process as a crucial step for mast cell degranulation and cytokine synthesis (123, 124). The connection between TGF-β levels and cell metabolism extends beyond allergic diseases. Sepsis, for instance, is linked to elevated circulating levels of TGF-β (125, 126). In line with this, glycolysis is the primary pathway determining the allocation of energy to organ cells during sepsis (127). The levels of TGF-β are elevated in joint synovial fluid of rheumatoid arthritis (RA) patients (128, 129). Glycolysis is increased in fibroblast-like synoviocytes in RA (130, 131).

In the cancer context, elevated TGF-β levels reprogram macrophage metabolism toward the glycolytic pathway while simultaneously suppressing their pro-inflammatory machinery (113) and diminishing their anti-cancer efficacy. Within adipose tissue, TGF-β-associated glycolysis in macrophages contributes to low-grade inflammation (132), a characteristic feature of obesity (133). Thus, the effect of TGF-β on both cancer cells and adipose tissue is, at least partially, mediated by macrophages and potentially other immune cells.

This data is presented in **Table 2**.

**Table 2:** The association between TGF-β levels or TGF-β/Smad signaling and metabolic bias (glycolysis vs. OXPHOS) in various diseases and in adipose tissue.

Condition/organ	TGF-β levels or signaling (tissue)	Ref.	Glyc./Oxph. (tissue/cells)	Ref.
Type 2 diabetes	↑ (serum and urine)	134 (a meta-analysis)	Glycolysis (pancreas β cells, liver, adipose tissue)	114
Asthma	↑ (serum)	16–19	Glycolysis (CD4+ T cells)	115
Neutrophilic Asthma	↑ (mice airways)	135	Glycolysis (sputum, nasal epithelium)	116
Atopic Dermatitis	↓ (buccal epithelium)	117	OXPHOS (nonlesional skin)	118
Atopic Dermatitis	↑ (AD lesions)	119	Glycolysis (a mouse model of lesional AD)	120
Urticaria	↓ (blood) (difference not statistically significant)	121	OXPHOS (mast cells)	123, 124
Sepsis	↑ (circulation)	125 (a mouse model), 126	Glycolysis (immune cells)	127
Rheumatoid arthritis	↑ (synovium)	128, 129	Glycolysis (fibroblast-like cynoviocytes)	130, 131
Advanced Solid Cancers	↑ (TME)	39	Glycolysis (macrophages)	113
Adipose Tissue	↑ (vs. lean subjects)	67, 136	Glycolysis (macrophages)	132

↑ = increased TGF-β levels or upregulated TGF-β-related gene expression (vs. healthy controls).

↓ = decreased TGF-β levels or downregulated TGF-β-related gene expression (vs. healthy controls).

Regarding cancer initiation, high levels of glucose uptake observed in poorly differentiated different types of cancer reflect persistence of the glycolytic metabolism of stem cells in malignant cells that fail to fully differentiate (137). Biasing cancer stem cells**’** metabolism toward the aerobic glycolytic pathway (the Warburg effect) drives their persistence in certain cancer types (breast cancer, nasopharyngeal carcinoma, hepatocellular carcinoma, uterine endometrial) (138, 139). As discussed above, TGF-β induces glycolysis and, therefore, promotes self-renewal in these cancers. However, stem cells of other cancer types (glioma, glioblastoma. lung cancer, pancreatic ductal adenocarcinoma, and leukemia) have demonstrated OXPHOS as their preferred energy production process (138, 139).

### The stage-dependent effect of TGF-β on the development of asthma is related to Th17 response

A clear distinction must be drawn between the association and causality of events. To establish causality, it is necessary for a change introduced in one event to result in some observable change in the second event. Reverting to the previous discussions, efforts have been undertaken to examine the causal relationship between TGF-β signaling and asthma. This has been pursued by studying the impact of anti-TGF-β/Smad agents on airway inflammation. There are indications that the role of TGF-β changes along the course of asthma development. TGF-β seems to inhibit the development of asthma in the initial stage of the disease, however it supports and maintains established inflammatory asthma. This temporal change in the role of TGF-β was reflected in the work by Fredriksson et al. (140) who demonstrated a contrasting effect of rapamycin on asthma which was depended on the stage of the disease. Rapamycin is known to activate the TGF-β signaling (141–143). Airway inflammation in a murine model was attenuated when rapamycin was administered during the induction stage of asthma, while inflammation was induced when rapamycin was administered in established asthma. In addition, rapamycin induced airway hyperactivity (i.e., exacerbation of the disease) in moderate lymphangioleiomyomatosis (a rare and progressive lung disease leading to the blockage of airways and blood vessels) (144). Here rapamycin was administered to patients with established lung inflammation. When CD4(+) T helper cells engineered to produce latent TGF-β1 were transferred to OVA-sensitized SCID mice 1-2 days *before* the mice were re-challenged with ovalbumin (OVA), airway inflammation was attenuated (145). Similarly, when TGF-β-secreting bone marrow stroma cells were injected intravenously to mice with ragweed induced asthma *at the time of the antigen challenge*, they protected the mice from most asthma-specific pathological changes, including Th2 related inflammatory cytokines and immunoglobulins (146).

Conversely, when nintedanib, a TGF-β/Smad pathway inhibitor (147), was given orally once a day (started day 35) to OVA-sensitized mice, *after* the starting of repeated OVA challenge (started day 31), airway inflammation was ameliorated (148). Similarly, when anti-TGF-β antibodies were injected to OVA-sensitized mice 30 minutes before each OVA-challenge for seven days, airway inflammation improved (149). Here again, the anti-TGF-β agent was administered (for 6 days) to mice after repeated OVA challenges. Pirfenidone is an antifibrotic agent that inhibits the TGF-β/Smad pathway (150). When OVA-sensitized mice were put on pirfenidone diet started before OVA-rechallenge and extended into a period of OVA-rechallenges, the results were mixed: pirfenidone had no effect on airway hyper-responsiveness, however pirfenidone significantly reduced distal bronchiolar cellularity and proximal and distal bronchiolar mucin content (151).

The pro-inflammatory role of TGF-β in *established* asthma may be related to asthma highly inflammatory environment that includes high concentrations of IL-6 and IL-21 (152), which in mice induce Th17 differentiation in the presence of low levels of TGF-β (31, 153) (in human, the presence of IL-1β is also required (32)). Th17 cytokines, including IL-17, play a role in neutrophilic inflammation in severe asthma (154) and IL-21is increased in human asthmatic airways (155). In the early stage of asthma, the concentrations of IL-6 and IL-21 are likely insufficient to induce Th17 differentiation. This inadequacy may explain the failure to reduce airway inflammation through TGF-β inhibition at this stage. Moreover, at this stage TGF-β suppresses the activity of the immune system with the result of reducing the inflammatory symptoms of asthma, observed in animal models of asthma.

It should be added that two studies demonstrate an opposite picture. Alcorn et al. (156) report that anti-TGF-β antibodies applied prior to each QVA challenge following a period of OVA sensitization, induced airway hypersensitivity *with no significant effect on airway inflammation i*n a mouse model. Similarly, McMillan et al. (157) have shown that anti-TGF-beta antibodies administered therapeutically, with dosing starting *after* the onset of established eosinophilic airway inflammation in mice, significantly reduced peribronchiolar extracellular matrix deposition, airway smooth muscle cell proliferation, and mucus production in the lung *without affecting established airway inflammation and Th2 cytokine production*. The contrasting results between different studies may be related to varying levels of TGF-β in each asthma model. As Zhou et al. (31) have shown, in mice, TGF-β orchestrates Th17 cell differentiation in a concentration-dependent manner. At low concentrations, TGF-β synergizes with IL-6 and IL-21 to promote IL-23R expression, favoring Th17 cell differentiation. This effect increases with TGF-β level and reaches an apex at a specific TGF-β level. TGF-β concentrations higher than this apex point repress IL-23R expression and favor Foxp3+ Treg cells. It should be remembered that at the same time, TGF-β suppresses Th2 (and Th1) cells (46) and inhibits mast cells activation (158), both involved in airway inflammation.

It is noted that a protective effect of TGF-β in early stage and a detrimental effect in late state on disease development is also typical to cancer (39, 159).

Overall, inhibiting TGF-β/Smad signaling seems to provide short-term benefits for patients with neutrophilic asthma, considering the established state of the disease. The question of potential serious adverse events associated with such treatment remains. However, the causative relationship between TGF-β/Smad signaling and long-term comorbidities is still unclear.

### Activin-A activity could be another factor contributing to the negative correlation between allergic rhinitis and the risk of certain types of cancer

The TGF-β superfamily member activin-A serves various biological functions and shares signaling pathways with TGF-β1. Ex vivo stimulations of nasal tissue from patients with nasal polyps have suggested a direct induction of each other’s expression at the mRNA level by activin-A and TGF-β1 (160). Additionally, recent findings indicate that Activin-A induces apoptosis of human lung adenocarcinoma A549 cells through the endoplasmic reticulum stress pathway (161). If this mechanism extends to other cancer types, it could elucidate the negative correlation between AR and the risk of these cancers. In contrast to the two mechanisms proposed earlier, this mechanism also predicts a beneficial effect of AR on cancer prognosis.

### Asthma patients with high TGF-β and Th17-related cytokine levels should be monitored for metabolic dysregulations and cancer

It is suggested here, that irrespective of the specific disease, allergy patients should individually be classified into two distinct groups: patients with high level of circulating TGF-β and Th17 cytokines and those with levels lower than or close to normal levels. The author hypothesizes that allergy patients belonging to the first group will display cell metabolism biased toward glycolysis. They will be more prone to develop obesity, insulin resistance, hypertension and DM2. These patients will also be subject to a higher risk of cancer compared with normal subjects. Patients belonging to the second group will display OXPHOS-biased cell metabolism, a lower association with metabolic dysregulations, and a reduced risk of cancer compared with normal subjects. Subjects belonging to the first group should be more closely watched for metabolic syndrome and cancer.

### The coexistence of asthma and allergic rhinitis

It is notable that asthma and AR often coexist. Estimates suggest that 19-38% of patients with AR also have concurrent asthma (162), while 85–95% of patients with asthma also experience AR (163). Drawing from the earlier discussions, the author posits that, in most cases, AR will tend to coexist more frequently with eosinophilic asthma and less so with neutrophilic asthma.

### Concluding remarks

The distinct inflammatory pathways of AR and neutrophilic asthma likely contribute to their contrasting associations with metabolic dysregulations and cancer risk. Given that TGF-β, IL-1β and IL-6 are essential for Th17 cell differentiation, the connection between neutrophilic asthma (characterized by high levels of these cytokines) and metabolic dysregulation and the risk of cancer becomes apparent. Specifically, in neutrophilic asthma, levels of IL-1β surpass those observed in eosinophilic asthma (notably, IL-6 levels in asthmatic patients do not correlate with the presence of neutrophils in the airways (164)). In comparison to AR, neutrophilic asthma displays higher values of both TGF-β and IL-1β circulating levels. The difference in cytokine levels between these two allergic conditions accounts for the opposing associations of neutrophilic asthma and allergic rhinitis with most types of cancer and metabolic dysregulations.

It is noteworthy that the involvement of TGF-β in metabolic dysregulations has been previously documented (165). Nevertheless, to the author**’**s knowledge, this is the first piece of work that establishes a connection between TGF-β/Smad pathway, IL-1β and IL-6 levels, metabolic pathways, the type of allergy, metabolic dysregulations, and the risk of cancer within a unified framework.

The schematic relationship between TGF-β levels, TGF-β signaling, metabolic pathways, metabolic dysregulations, allergic rhinitis and cancer risk, is depicted in **Figure 1**.

## Summary

The current study proposes a binary classification of TGF-β/Smad pathway activity as either high or low, aiming to elucidate the divergent connections between various allergic diseases and the risk of cancer, as well as their contrasting association with metabolic dysregulations. High levels of TGF-β and Th17 inflammatory cytokines in neutrophilic asthma, and low levels of TGF-β in allergic rhinitis may provide an explanation for the differences in long-term comorbidities between neutrophilic asthma and allergic rhinitis. The binary nature of these relationships is suggested to be governed by the two metabolic pathways of eukaryotic cells: glycolysis and oxidative phosphorylation.

## Data availability statement

The original contributions presented in the study are included in the article/supplementary material. Further inquiries can be directed to the corresponding author.

## Author contributions

ZE: Writing – review & editing, Writing – original draft, Validation, Supervision, Methodology, Investigation, Data curation, Conceptualization.

## References

[1] Buelow B Routes JM. Immediate hypersensitivity reaction. Medscape. (2020). Available at: https://emedicine.medscape.com/article/136217-overview?form=fpf#a5.

[2] Hemminki K Försti A Fallah M Sundquist J Sundquist K Ji J. Risk of cancer in patients with medically diagnosed hay fever or allergic rhinitis. Int J Cancer. (2014) 135:2397–403. doi: 10.1002/ijc.28873 24692097

[3] Liu X Hemminki K Försti A Sundquist J Sundquist K Ji J. Cancer risk and mortality in asthma patients: A Swedish national cohort study. Acta Oncol. (2015) 54:1120–7. doi: 10.3109/0284186X.2014.1001497 25608824

[4] Wondmkun YT. Obesity, insulin resistance, and type 2 diabetes: associations and therapeutic implications. Diabetes Metab Syndr Obes. (2020) 13:3611–6. doi: 10.2147/DMSO.S275898 PMC7553667 33116712

[5] Torres RM Souza MDS Coelho ACC de Mello LM Souza-MaChado C. Association between asthma and type 2 diabetes mellitus: mechanisms and impact on asthma control-A literature review. Can Respir J. (2021) 2021:8830439. doi: 10.1155/2021/8830439 33520042 PMC7817304

[6] Zolotareva O Saik OV Königs C Bragina EY Goncharova IA Freidin MB. Comorbidity of asthma and hypertension may be mediated by shared genetic dysregulation and drug side effects. Sci Rep. (2019) 9:16302. doi: 10.1038/s41598-019-52762-w 31705029 PMC6841742

[7] Jiang D Wang L Bai C Chen O. Association between abdominal obesity and asthma: a meta-analysis. Allergy Asthma Clin Immunol. (2019) 15:16. doi: 10.1186/s13223-019-0333-6 30949213 PMC6431003

[8] Shore SA. Obesity and asthma: lessons from animal models. J Appl Physiol (1985). (2007) 102:516–28. doi: 10.1152/japplphysiol.00847.2006 17053103

[9] Kong J Yang F Bai M Zong Y Li Z Meng X. Airway immune response in the mouse models of obesity-related asthma. Front Physiol. (2022) 13:909209. doi: 10.3389/fphys.2022.909209 36051916 PMC9424553

[10] Daniels Gatward LF Kennard MR Smith LIF King AJF. The use of mice in diabetes research: The impact of physiological characteristics, choice of model and husbandry practices. Diabetes Med. (2021) 38:e14711. doi: 10.1111/dme.14711 34614258

[11] Gu C Loube J Lee R Bevans-Fonti S Wu TD Barmine JH. Metformin alleviates airway hyperresponsiveness in a mouse model of diet-induced obesity. Front Physiol. (2022) 13:883275. doi: 10.3389/fphys.2022.883275 35574481 PMC9098833

[12] Lee TK Jeon YJ Jung SJ. Bi-directional association between allergic rhinitis and diabetes mellitus from the national representative data of South Korea. Sci Rep. (2021) 11:4344. doi: 10.1038/s41598-021-83787-9 33623055 PMC7902822

[13] Hashimoto Y Futamura A. Prevalence of allergic rhinitis is lower in subjects with higher levels of fasting plasma glucose. Diabetes Care. (2010) 33:e143. doi: 10.2337/dc10-1338 20980422

[14] Hwang IC Lee YJ Ahn HY Lee SM. Association between allergic rhinitis and metabolic conditions: a nationwide survey in Korea. Allergy Asthma Clin Immunol. (2016) 12:5. doi: 10.1186/s13223-015-0108-7 26807136 PMC4722723

[15] Han YY Forno E Gogna M Celedón JC. Obesity and rhinitis in a nationwide study of children and adults in the United States. J Allergy Clin Immunol. (2016) 137:1460–5. doi: 10.1016/j.jaci.2015.12.1307 PMC4860058 26883461

[16] Manuyakorn W Kamchaisatian W Atamasirikul K Sasisakulporn C Direkwattanachai C Benjaponpitak S. Serum TGF-beta1 in atopic asthma. Asian Pac J Allergy Immunol. (2008) 26:185–9. 19317336

[17] Ozyilmaz E Canbakan S Capan N Erturk A Gulhan M. Correlation of plasma transforming growth factor beta 1 with asthma control test. Allergy Asthma Proc. (2009) 30:35–40. doi: 10.2500/aap.2009.30.3192 19331718

[18] Karagiannidis C Hense G Martin C Epstein M Rückert B Mantel PY. Activin A is an acute allergen-responsive cytokine and provides a link to TGF-beta-mediated airway remodeling in asthma. J Allergy Clin Immunol. (2006) 117:111–8. doi: 10.1016/j.jaci.2005.09.017 16387593

[19] Jiang K Chen HB Wang Y Lin JH Hu Y Fang YR. Changes in interleukin-17 and transforming growth factor beta 1 levels in serum and bronchoalveolar lavage fluid and their clinical significance among children with asthma. Transl Pediatr. (2013) 2:154–9. doi: 10.3978/j.issn.2224-4336.2013.10.03 PMC4729083 26835311

[20] Rosas A Valencia MP Sánchez M Bautista M Rico G Vega GB. Transforming growth factor beta and platelets in allergic rhinitis and sinusitis. Rev Alerg Mex. (2011) 58:93–8. 21967968

[21] Chai SK Altman GM Yazdanbakhsh M Tsuji J Godat L Takaro TK. Production of interleukin 10 and transforming growth factor beta in concomitant allergy and autoimmunity. Ann Allergy Asthma Immunol. (2005) 94:279–85. doi: 10.1016/S1081-1206(10)61309-9 15765746

[22] Lee SS Won TB Kim JW Rhee CS Lee CH Hong SC. Effects of dexamethasone on the expression of transforming growth factor-beta in the mouse model of allergic rhinitis. Laryngoscope. (2007) 117:1323–8. doi: 10.1097/MLG.0b013e318064e84d 17762268

[23] Ciprandi G De Amici M Tosca M Marseglia G. Serum transforming growth factor-beta levels depend on allergen exposure in allergic rhinitis. Int Arch Allergy Immunol. (2010) 152:66–70. doi: 10.1159/000260085 19940507

[24] Qiu Q Lu H Lu C Chen S Han H. Variations in TGF-beta, IL-10, and IL-17 after specific immunotherapy and correlations with symptoms in patients with allergic rhinitis. J Investig Allergol Clin Immunol. (2012) 22:311–2. 22812212

[25] Mirzaei Y Savari Z Yazdani-Nafchi F Salehi-Vanani N Fallahi E Pirayesh A. The expression analysis of IL-6, IL-18, IL-21, IL-23, and TGF-β mRNA in the nasal mucosa of patients with Allergic rhinitis. Afr Health Sci. (2022) 22:630–40. doi: 10.4314/ahs.v22i1.73 PMC9382521 36032502

[26] Bayrak Degirmenci P Aksun S Altin Z Bilgir F Arslan IB Colak H. Allergic rhinitis and its relationship with IL-10, IL-17, TGF-β, IFN-γ, IL 22, and IL-35. Dis Markers. (2018) 2018:9131432. doi: 10.1155/2018/9131432 29692871 PMC5859791

[27] Hammad H Lambrecht BN. The basic immunology of asthma. Cell. (2021) 184:1469–85. doi: 10.1016/j.cell.2021.02.016 33711259

[28] Maison N Omony J Illi S Thiele D Skevaki C Dittrich AM. T2-high asthma phenotypes across lifespan. Eur Respir J. (2022) 60:2102288. doi: 10.1183/13993003.02288-2021 35210326 PMC9520028

[29] Das S Miller M Broide DH. Chromosome 17q21 genes ORMDL3 and GSDMB in asthma and immune diseases. Adv Immunol. (2017) 135:1–52. doi: 10.1016/bs.ai.2017.06.001 28826527

[30] Ciprandi G Cuppari C Salpietro A Tosca M Grasso L Rigoli L. Serum IL-23 in asthmatic children. J Biol Regul Homeost Agents. (2012) 26:S53–61. 22691251

[31] Zhou L Lopes JE Chong MM Ivanov II Min R Victora GD. TGF-beta-induced Foxp3 inhibits T(H)17 cell differentiation by antagonizing RORgammat function. Nature. (2008) 453:236–40. doi: 10.1038/nature06878 PMC2597437 18368049

[32] Manel N Unutmaz D Littman DR. The differentiation of human T(H)-17 cells requires transforming growth factor-beta and induction of the nuclear receptor RORgammat. Nat Immunol. (2008) 9:641–9. doi: 10.1038/ni.1610 PMC2597394 18454151

[33] Lluis A Schedel M Liu J Illi S Depner M von Mutius E. Asthma-associated polymorphisms in 17q21 influence cord blood ORMDL3 and GSDMA gene expression and IL-17 secretion. J Allergy Clin Immunol. (2011) 127:1587–94.e6. doi: 10.1016/j.jaci.2011.03.015 21546069

[34] Bantulà M Roca-Ferrer J Arismendi E Picado C. Asthma and obesity: two diseases on the rise and bridged by inflammation. J Clin Med. (2021) 10:169. doi: 10.3390/jcm10020169 33418879 PMC7825135

[35] Guma M Ronacher L Liu-Bryan R Takai S Karin M Corr M. Caspase 1-independent activation of interleukin-1beta in neutrophil-predominant inflammation. Arthritis Rheumatol. (2009) 60:3642–50. doi: 10.1002/art.24959 PMC2847793 19950258

[36] Prame Kumar K Nicholls AJ Wong CHY. Partners in crime: neutrophils and monocytes/macrophages in inflammation and disease. Cell Tissue Res. (2018) 371:551–65. doi: 10.1007/s00441-017-2753-2 PMC5820413 29387942

[37] Chang C Chen G Wu W Chen D Chen S Gao J. Exogenous IL-25 ameliorates airway neutrophilia *via* suppressing macrophage M1 polarization and the expression of IL-12 and IL-23 in asthma. Respir Res. (2023) 24:260. doi: 10.1186/s12931-023-02557-5 37898756 PMC10613395

[38] Simpson JL Phipps S Baines KJ Oreo KM Gunawardhana L Gibson PG. Elevated expression of the NLRP3 inflammasome in neutrophilic asthma. Eur Respir J. (2014) 43:1067–76. doi: 10.1183/09031936.00105013 24136334

[39] Elkoshi Z. Cancer and autoimmune diseases: A tale of two immunological opposites? Front Immunol. (2022) 25:821598. doi: 10.3389/fimmu.2022.821598 PMC8822211 35145524

[40] Shevyrev D Tereshchenko V. Treg heterogeneity, function, and homeostasis. Front Immunol. (2020) 10:3100. doi: 10.3389/fimmu.2019.03100 31993063 PMC6971100

[41] Veglia F Sanseviero E Gabrilovich DI. Myeloid-derived suppressor cells in the era of increasing myeloid cell diversity. Nat Rev Immunol. (2021) 21:485–98. doi: 10.1038/s41577-020-00490-y PMC7849958 33526920

[42] Kalathil SG Thanavala Y. Importance of myeloid derived suppressor cells in cancer from a biomarker perspective. Cell Immunol. (2021) 361:104280. doi: 10.1016/j.cellimm.2020.104280 33445053 PMC9204650

[43] Fridlender ZG Sun J Kim S Kapoor V Cheng G Ling L. Polarization of tumor-associated neutrophil phenotype by TGF-beta: "N1" versus "N2" TAN. Cancer Cell. (2009) 16:183–94. doi: 10.1016/j.ccr.2009.06.017 PMC2754404 19732719

[44] Zhang F Wang H Wang X Jiang G Liu H Zhang G. TGF-β induces M2-like macrophage polarization *via* SNAIL-mediated suppression of a pro-inflammatory phenotype. Oncotarget. (2016) 7:52294–306. doi: 10.18632/oncotarget.10561 PMC5239552 27418133

[45] Schmidt A Éliás S Joshi RN Tegnér J. *In vitro* differentiation of human CD4+FOXP3+ Induced regulatory T cells (iTregs) from naïve CD4+ T cells using a TGF-β-containing protocol. J Vis Exp. (2016) 118):55015. doi: 10.3791/55015 PMC5226637 28060341

[46] Chen W. TGF-β Regulation of T cells. Annu Rev Immunol. (2023) 41:483–512. doi: 10.1146/annurev-immunol-101921-045939 36750317 PMC12453633

[47] Cao P Sun Z Zhang F Zhang J Zheng X Yu B. TGF-β Enhances immunosuppression of myeloid-derived suppressor cells to induce transplant immune tolerance through affecting arg-1 expression. Front Immunol. (2022) 13:919674. doi: 10.3389/fimmu.2022.919674 35874674 PMC9300822

[48] Greten FR Grivennikov SI. Inflammation and cancer: triggers, mechanisms, and consequences. Immunity. (2019) 51:27–41. doi: 10.1016/j.immuni.2019.06.025 31315034 PMC6831096

[49] Peñuelas S Anido J Prieto-Sánchez RM Folch G Barba I Cuartas I. TGF-beta increases glioma-initiating cell self-renewal through the induction of LIF in human glioblastoma. Cancer Cell. (2009) 15:315–27. doi: 10.1016/j.ccr.2009.02.011 19345330

[50] Nana AW Yang PM Lin HY. Overview of transforming growth factor β Superfamily involvement in glioblastoma initiation and progression. Asian Pac J Cancer Prev. (2015) 16:6813–23. doi: 10.7314/apjcp.2015.16.16.6813 26514451

[51] Nie Z Wang C Zhou Z Chen C Liu R Wang D. Transforming growth factor-beta increases breast cancer stem cell population partially through upregulating PMEPA1 expression. Acta Biochim Biophys Sin (Shanghai). (2016) 48:194–201. doi: 10.1093/abbs/gmv130 26758191

[52] Futakuchi M Lami K Tachibana Y Yamamoto Y Furukawa M Fukuoka J. The effects of TGF-β Signaling on cancer cells and cancer stem cells in the bone microenvironment. Int J Mol Sci. (2019) 20:5117. doi: 10.3390/ijms20205117 31619018 PMC6829436

[53] Lonardo E Hermann PC Mueller MT Huber S Balic A Miranda-Lorenzo I. Nodal/Activin signaling drives self-renewal and tumorigenicity of pancreatic cancer stem cells and provides a target for combined drug therapy. Cell Stem Cell. (2011) 9:433–46. doi: 10.1016/j.stem.2011.10.001 22056140

[54] Song K Wang H Krebs TL Kim SJ Danielpour D. Androgenic control of transforming growth factor-beta signaling in prostate epithelial cells through transcriptional suppression of transforming growth factor-beta receptor II. Cancer Res. (2008) 68:8173–82. doi: 10.1158/0008-5472.CAN-08-2290 PMC2596934 18829577

[55] Ito I Hanyu A Wayama M Goto N Katsuno Y Kawasaki S. Estrogen inhibits transforming growth factor beta signaling by promoting Smad2/3 degradation. J Biol Chem. (2010) 285:14747–55. doi: 10.1074/jbc.M109.093039 PMC2863224 20207742

[56] Hernández-Vega AM Camacho-Arroyo I. Crosstalk between 17β-estradiol and TGF-β Signaling modulates glioblastoma progression. Brain Sci. (2021) 11:564. doi: 10.3390/brainsci11050564 33925221 PMC8145480

[57] Kunzmann S Ottensmeier B Speer CP Fehrholz M. Effect of progesterone on Smad signaling and TGF-β/Smad-regulated genes in lung epithelial cells. PloS One. (2018) 13:e0200661. doi: 10.1371/journal.pone.0200661 30001393 PMC6042760

[58] Lindgren PR Bäckström T Cajander S Damber MG Mählck CG Zhu D. The pattern of estradiol and progesterone differs in serum and tissue of benign and Malignant ovarian tumors. Int J Oncol. (2002) 21(3):583–9. doi: 10.3892/ijo 12168103

[59] Rodriguez AC Blanchard Z Maurer KA Gertz J. Estrogen signaling in endometrial cancer: a key oncogenic pathway with several open questions. Horm Cancer. (2019) 10:51–63. doi: 10.1007/s12672-019-0358-9 30712080 PMC6542701

[60] Zouboulis CC Chen WC Thornton MJ Qin K Rosenfield R. Sexual hormones in human skin. Horm Metab Res. (2007) 39:85–95. doi: 10.1055/s-2007-961807 17326004

[61] Parikh R Sorek E Parikh S Michael K Bikovski L Tshori S. Skin exposure to UVB light induces a skin-brain-gonad axis and sexual behavior. Cell Rep. (2021) 36:109579. doi: 10.1016/j.celrep.2021.109579 34433056 PMC8411113

[62] Ernster VL Wrensch MR Petrakis NL King EB Miike R Murai J. Benign and Malignant breast disease: initial study results of serum and breast fluid analyses of endogenous estrogens. J Natl Cancer Inst. (1987) 79:949–60. 3479643

[63] Samoli E Trichopoulos D Lagiou A Zourna P Georgila C Minaki P. The hormonal profile of benign breast disease. Br J Cancer. (2013) 108:199–204. doi: 10.1038/bjc.2012.493 23169293 PMC3553510

[64] Visscher DW Frost MH Hartmann LC Frank RD Vierkant RA McCullough AE. Clinicopathologic features of breast cancers that develop in women with previous benign breast disease. Cancer. (2016) 122:378–85. doi: 10.1002/cncr.29766 PMC4724320 26512815

[65] Salib RJ Kumar S Wilson SJ Howarth PH. Nasal mucosal immunoexpression of the mast cell chemoattractants TGF-beta, eotaxin, and stem cell factor and their receptors in allergic rhinitis. J Allergy Clin Immunol. (2004) 114:799–806. doi: 10.1016/j.jaci.2004.07.010 15480318

[66] Gosset P Malaquin F Delneste Y Wallaert B Capron A Mählck CG. Interleukin-6 and interleukin-1 alpha production is associated with antigen-induced late nasal response. J Allergy Clin Immunol. (1993) 92:878–90. doi: 10.1016/0091-6749(93)90066-O 8258622

[67] Yadav H Quijano C Kamaraju AK Gavrilova O Malek R Chen W. Protection from obesity and diabetes by blockade of TGF-β/Smad3 signaling. Cell Metab. (2011) 14:67–79. doi: 10.1016/j.cmet.2011.04.013 21723505 PMC3169298

[68] Lee MJ. Transforming growth factor beta superfamily regulation of adipose tissue biology in obesity. Biochim Biophys Acta Mol Basis Dis. (2018) 1864:1160–71. doi: 10.1016/j.bbadis.2018.01.025 29409985

[69] El-Gohary Y Tulachan S Wiersch J Guo P Welsh C Prasadan K. A smad signaling network regulates islet cell proliferation. Diabetes. (2014) 63:224–36. doi: 10.2337/db13-0432 PMC3868054 24089514

[70] Sheng J Wang L Tang PM Wang HL Li JC Xu BH. Smad3 deficiency promotes beta cell proliferation and function in db/db mice *via* restoring Pax6 expression. Theranostics. (2021) 11:2845–59. doi: 10.7150/thno.51857 PMC7806493 33456576

[71] Wang HL Wang L Zhao CY Lan HY. Role of TGF-beta signaling in beta cell proliferation and function in diabetes. Biomolecules. (2022) 12:373. doi: 10.3390/biom12030373 35327565 PMC8945211

[72] Rajendran S Anquetil F Quesada-Masachs E Graef M Gonzalez N McArdle S. IL-6 is present in beta and alpha cells in human pancreatic islets: Expression is reduced in subjects with type 1 diabetes. Clin Immunol. (2020) 211:108320. doi: 10.1016/j.clim.2019.108320 31809899 PMC6961707

[73] Anquetil F Sabouri S Thivolet C Rodriguez-Calvo T Zapardiel-Gonzalo J Amirian N. Alpha cells, the main source of IL-1β in human pancreas. J Autoimmun. (2017) 81:68–73. doi: 10.1016/j.jaut.2017.03.006 28325643 PMC5507672

[74] Porreca E Di Febbo C Mincione G Reale M Baccante G Guglielmi MD. Increased transforming growth factor-beta production and gene expression by peripheral blood monocytes of hypertensive patients. Hypertension. (1997) 30:134–9. doi: 10.1161/01.hyp.30.1.134 9231833

[75] Lijnen PJ Petrov VV Fagard RH. Association between transforming growth factor-beta and hypertension. Am J Hypertens. (2003) 16:604–11. doi: 10.1016/s0895-7061(03)00847-1 12850397

[76] Tanase DM Gosav EM Radu S Ouatu A Rezus C Ciocoiu M. Arterial hypertension and interleukins: potential therapeutic target or future diagnostic marker? Int J Hypertens. (2019) 2019:3159283. doi: 10.1155/2019/3159283 31186952 PMC6521461

[77] Kang N Song WJ. Discovering biomarkers of neutrophilic asthma: A clinician's perspective. Allergy Asthma Immunol Res. (2022) 14:1–4. doi: 10.4168/aair.2022.14.1.1 34983102 PMC8724821

[78] Wang HR Wei SZ Song XY Wang Y Zhang WB Ren C. IL-1β and allergy: focusing on its role in allergic rhinitis. Mediators Inflamm. (2023) 2023:1265449. doi: 10.1155/2023/1265449 37091903 PMC10115535

[79] Han MW Kim SH Oh I Kim YH Lee J. Serum IL-1β can be a biomarker in children with severe persistent allergic rhinitis. Allergy Asthma Clin Immunol. (2019) 15:58. doi: 10.1186/s13223-019-0368-8 31548841 PMC6749717

[80] Gao S Yu L Zhang J Li X Zhou J Zeng P. Expression and clinical significance of VCAM-1, IL-6, and IL-17A in patients with allergic rhinitis. Ann Palliat Med. (2021) 10:4516–22. doi: 10.21037/apm-21-546 33966399

[81] Recalde M Pistillo A Davila-Batista V Leitzmann M Romieu I Viallon V. Longitudinal body mass index and cancer risk: a cohort study of 2.6 million Catalan adults. Nat Commun. (2023) 14:3816. doi: 10.1038/s41467-023-39282-y 37391446 PMC10313757

[82] Pothiawala S Qureshi AA Li Y Han J. Obesity and the incidence of skin cancer in US Caucasians. Cancer Causes Control. (2012) 23:717–26. doi: 10.1007/s10552-012-9941-x PMC3704194 22450736

[83] Bjørge T Lie AK Engeland A. The impact of height and body mass index on the risk of testicular cancer in 600,000 Norwegian men. Cancer Causes Control. (2006) 17:983–7. doi: 10.1007/s10552-006-0032-8 16841265

[84] Davies TW Prener A Engholm G. Body size and cancer of the testis. Acta Oncol. (1990) 29:287–90. doi: 10.3109/02841869009089999 2363939

[85] Wise MR Jordan V Lagas A Showell M Wong N Lensen S. Obesity and endometrial hyperplasia and cancer in premenopausal women: A systematic review. Am J Obstet Gynecol. (2016) 214:689.e1–689.e17. doi: 10.1016/j.ajog.2016.01.175 26829507

[86] Foong KW Bolton H. Obesity and ovarian cancer risk: A systematic review. Post Reprod Health. (2017) 23:183–98. doi: 10.1177/2053369117709225 28720017

[87] Telenga ED Tideman SW Kerstjens HA Hacken NH Timens W Postma DS. Obesity in asthma: more neutrophilic inflammation as a possible explanation for a reduced treatment response. Allergy. (2012) 67:1060–8. doi: 10.1111/j.1398-9995.2012.02855.x 22686834

[88] Scott HA Gibson PG Garg ML Wood LG. Airway inflammation is augmented by obesity and fatty acids in asthma. Eur Respir J. (2011) 38:594–602. doi: 10.1183/09031936.00139810 21310876

[89] Haldar P Pavord ID Shaw DE Berry MA Thomas M Brightling CE. Cluster analysis and clinical asthma phenotypes. Am J Respir Crit Care Med. (2008) 178:218–24. doi: 10.1164/rccm.200711-1754OC PMC3992366 18480428

[90] Todd DC Armstrong S D'Silva L Allen CJ Hargreave FE Parameswaran K. Effect of obesity on airway inflammation: a cross-sectional analysis of body mass index and sputum cell counts. Clin Exp Allergy. (2007) 37:1049–54. doi: 10.1111/j.1365-2222.2007.02748.x 17581198

[91] Rhee H Love T Harrington D. Blood neutrophil count is associated with body mass index in adolescents with asthma. JSM Allergy Asthma. (2018) 3:1019. 30542672 PMC6287916

[92] Wang Y Wan R Hu C. Leptin/obR signaling exacerbates obesity-related neutrophilic airway inflammation through inflammatory M1 macrophages. Mol Med. (2023) 29:100. doi: 10.1186/s10020-023-00702-w 37488474 PMC10367413

[93] Uribe-Querol E Rosales C. Neutrophils actively contribute to obesity-associated inflammation and pathological complications. Cells. (2022) 11:1883. doi: 10.3390/cells11121883 35741012 PMC9221045

[94] Gutmann D Dressler M Eickmeier O Herrmann E Kirwil M Schubert R. Proinflammatory pattern in the lower airways of non-asthmatic obese adolescents. Cytokine. (2024) 173:156452. doi: 10.1016/j.cyto.2023.156452 38039695

[95] Amin K Issa SM Ali KM Aziz MI Hama Amieen HM Bystrom J. Evidence for eosinophil and IL-17 mediated inflammation in allergic rhinitis. Clin Mol Allergy. (2020) 18:6. doi: 10.1186/s12948-020-00117-6 32280308 PMC7129325

[96] Qing M Yongge L Wei X Yan W Zhen L Yixin R. Comparison of Th17 cells mediated immunological response among asthmatic children with or without allergic rhinitis. Asian Pac J Allergy Immunol. (2019) 37:65–72. doi: 10.12932/AP-140317-0047 29602286

[97] Shirkani A Mansouri A Farid Hosseini R Jabbari Azad F Alsadat Mahmoudian R Montazer M. The role of interleukin-4 and 13 gene polymorphisms in allergic rhinitis: A case control study. Rep Biochem Mol Biol. (2019) 8:111–8. PMC6844616 31832433

[98] Le Floc'h A Allinne J Nagashima K Scott G Birchard D Asrat S. Dual blockade of IL-4 and IL-13 with dupilumab, an IL-4Rα antibody, is required to broadly inhibit type 2 inflammation. Allergy. (2020) 75:1188–204. doi: 10.1111/all.14151 PMC7317958 31838750

[99] Campion NJ Doralt A Lupinek C Berger M Poglitsch K Brugger J. Dupilumab reduces symptom burden in allergic rhinitis and suppresses allergen-specific IgE production. Allergy. (2023) 78:1687–91. doi: 10.1111/all.15653 36691369

[100] Gorelik L Fields PE Flavell RA. Cutting edge: TGF-beta inhibits Th type 2 development through inhibition of GATA-3 expression. J Immunol. (2000) 165:4773–7. doi: 10.4049/jimmunol.165.9.4773 11045997

[101] Al-Alawi M Hassan T Chotirmall SH. Transforming growth factor β and severe asthma: a perfect storm. Respir Med. (2014) 108:1409–23. doi: 10.1016/j.rmed.2014.08.008 25240764

[102] Zou XL Chen ZG Zhang TT Feng DY Li HT Yang HL. Th17/Treg homeostasis, but not Th1/Th2 homeostasis, is implicated in exacerbation of human bronchial asthma. Ther Clin Risk Manage. (2018) 14:1627–36. doi: 10.2147/TCRM.S172262 PMC6132476 30233198

[103] Elkoshi Z. The binary classification of chronic diseases. J Inflammation Res. (2019) 12:319–33. doi: 10.2147/JIR.S227279 PMC6927256 31908517

[104] Elkoshi Z. The binary model of chronic diseases applied to COVID-19. Front Immunol. (2021) 12:716084. doi: 10.3389/fimmu.2021.716084 34539649 PMC8446604

[105] Elkoshi Z. SARS-coV-2 omicron (B.1.1.529) variant: corticosteroids treatment/respiratory coinfection. Front Immunol. (2022) 13:856072. doi: 10.3389/fimmu.2022.856072 35309339 PMC8927656

[106] Geeraerts X Bolli E Fendt SM Van Ginderachter JA. Macrophage metabolism as therapeutic target for cancer, atherosclerosis, and obesity. Front Immunol. (2017) 8:289. doi: 10.3389/fimmu.2017.00289 28360914 PMC5350105

[107] Soto-Heredero G Gómez de Las Heras MM Gabandé-Rodríguez E Oller J Mittelbrunn M. Glycolysis - a key player in the inflammatory response. FEBS J. (2020) 287:3350–69. doi: 10.1111/febs.15327 PMC7496292 32255251

[108] Lee SY Jeon HM Ju MK Jeong EK Kim CH Yoo MA. Dlx-2 is implicated in TGF-β- and Wnt-induced epithelial-mesenchymal, glycolytic switch, and mitochondrial repression by Snail activation. Int J Oncol. (2015) 46:1768–80. doi: 10.3892/ijo.2015.2874 25651912

[109] Zhang D Wang Y Shi Z Liu J Sun P Hou X. Metabolic reprogramming of cancer-associated fibroblasts by IDH3α downregulation. Cell Rep. (2015) 10:1335–48. doi: 10.1016/j.celrep.2015.02.006 25732824

[110] Rodríguez-García A Samsó P Fontova P Simon-Molas H Manzano A Castaño E. TGF-β1 targets Smad, p38 MAPK, and PI3K/Akt signaling pathways to induce PFKFB3 gene expression and glycolysis in glioblastoma cells. FEBS J. (2017) 284:3437–54. doi: 10.1111/febs.14201 28834297

[111] Hu ML Wang XY Chen WM. TGF-β1 upregulates the expression of lncRNA UCA1 and its downstream HXK2 to promote the growth of hepatocellular carcinoma. Eur Rev Med Pharmacol Sci. (2018) 22:4846–54. doi: 10.26355/eurrev_201808_15620 30070319

[112] Wang C Silverman RM Shen J O'Keefe RJ. Distinct metabolic programs induced by TGF-β1 and BMP2 in human articular chondrocytes with osteoarthritis. J Orthop Translat. (2018) 12:66–73. doi: 10.1016/j.jot.2017.12.004 29662780 PMC5866480

[113] Gauthier T Yao C Dowdy T Jin W Lim YJ Patiño LC. TGF-β uncouples glycolysis and inflammation in macrophages and controls survival during sepsis. Sci Signal. (2023) 16:eade0385. doi: 10.1126/scisignal.ade0385 37552767 PMC11145950

[114] Guo X Li H Xu H Woo S Dong H Lu F. Glycolysis in the control of blood glucose homeostasis. Acta Pharm Sin B. (2012) 2:358–67. doi: 10.1016/j.apsb.2012.06.002

[115] Ostroukhova M Goplen N Karim MZ Michalec L Guo L Liang Q. The role of low-level lactate production in airway inflammation in asthma. Am J Physiol Lung Cell Mol Physiol. (2012) 302:L300–7. doi: 10.1152/ajplung.00221.2011 PMC3289274 22080752

[116] Qian X Aboushousha R van de Wetering C Chia SB Amiel E Schneider RW. IL-1/inhibitory κB kinase ϵ-induced glycolysis augment epithelial effector function and promote allergic airways disease. J Allergy Clin Immunol. (2018) 142:435–450.e10. doi: 10.1016/j.jaci.2017.08.043 29108965 PMC6278819

[117] Arkwright PD Chase JM Babbage S Pravica V David TJ Hutchinson IV. Atopic dermatitis is associated with a low-producer transforming growth factor beta(1) cytokine genotype. J Allergy Clin Immunol. (2001) 108:281–4. doi: 10.1067/mai.2001.117259 11496247

[118] Leman G Pavel P Hermann M Crumrine D Elias PM Minzaghi D. Mitochondrial activity is upregulated in nonlesional atopic dermatitis and amenable to therapeutic intervention. J Invest Dermatol. (2022) 142(10):2623–2634.e12. doi: 10.1016/j.jid.2022.01.035 35341734

[119] Toda M Leung DY Molet S Boguniewicz M Taha R Christodoulopoulos P. Polarized in *vivo* expression of IL-11 and IL-17 between acute and chronic skin lesions. J Allergy Clin Immunol. (2003) 111:875–81. doi: 10.1067/mai.2003.1414 12704372

[120] Pavel P Leman G Hermann M Ploner C Eichmann TO Minzaghi D. Peroxisomal fatty acid oxidation and glycolysis are triggered in mouse models of lesional atopic dermatitis. JID Innov. (2021) 1:100033. doi: 10.1016/j.xjidi.2021.100033 34909730 PMC8659757

[121] Degirmenci PB Kırmaz C Vatansever S Onur E Nal E Erdin S. Analysis of the association of chronic spontaneous urticaria with interlekin-4, -10, transforming growth factor-β1, interferon-γ, interleukin-17A and -23 by autologous serum skin test. Postepy Dermatol Alergol. (2017) 34:70–6. doi: 10.5114/pdia.2016.57679 PMC5329100 28261034

[122] Church MK Kolkhir P Metz M Maurer M. The role and relevance of mast cells in urticaria. Immunol Rev. (2018) 282:232–47. doi: 10.1111/imr.12632 29431202

[123] Erlich TH Yagil Z Kay G Peretz A Migalovich-Sheikhet H Tshori S. Mitochondrial STAT3 plays a major role in IgE-antigen-mediated mast cell exocytosis. J Allergy Clin Immunol. (2014) 134:460–9. doi: 10.1016/j.jaci.2013.12.1075 24582310

[124] Chelombitko MA Chernyak BV Fedorov AV Zinovkin RA Razin E Paruchuru LB. The role played by mitochondria in fcϵRI-dependent mast cell activation. Front Immunol. (2020) 16:584210. doi: 10.3389/fimmu.2020.584210 PMC7596649 33178217

[125] Ahmad S Choudhry MA Shankar R Sayeed MM. Transforming growth factor-beta negatively modulates T-cell responses in sepsis. FEBS Lett. (1997) 402:213–8. doi: 10.1016/s0014-5793(96)01535-9 9037198

[126] Miller-Graziano CL Szabo G Griffey K Mehta B Kodys K Catalano D. Role of elevated monocyte transforming growth factor beta (TGF beta) production in posttrauma immunosuppression. J Clin Immunol. (1991) 11:95–102. doi: 10.1007/BF00917745 1905306

[127] Liu J Zhou G Wang X Liu D. Metabolic reprogramming consequences of sepsis: adaptations and contradictions. Cell Mol Life Sci. (2022) 79:456. doi: 10.1007/s00018-022-04490-0 35904600 PMC9336160

[128] Cheon H Yu SJ Yoo DH Chae IJ Song GG Sohn J. Increased expression of pro-inflammatory cytokines and metalloproteinase-1 by TGF-beta1 in synovial fibroblasts from rheumatoid arthritis and normal individuals. Clin Exp Immunol. (2002) 127:547–52. doi: 10.1046/j.1365-2249.2002.01785.x PMC1906321 11966774

[129] Gonzalo-Gil E Criado G Santiago B Dotor J Pablos JL Galindo M. Transforming growth factor (TGF)-β signalling is increased in rheumatoid synovium but TGF-β blockade does not modify experimental arthritis. Clin Exp Immunol. (2013) 174:245–55. doi: 10.1111/cei.12179 PMC3828828 23869798

[130] de Oliveira PG Farinon M Sanchez-Lopez E Miyamoto S Guma M. Fibroblast-like synoviocytes glucose metabolism as a therapeutic target in rheumatoid arthritis. Front Immunol. (2019) 10:1743. doi: 10.3389/fimmu.2019.01743 31428089 PMC6688519

[131] Garcia-Carbonell R Divakaruni AS Lodi A Vicente-Suarez I Saha A Cheroutre H. Critical role of glucose metabolism in rheumatoid arthritis fibroblast-like synoviocytes. Arthritis Rheumatol. (2016) 68:1614–26. doi: 10.1002/art.39608 PMC4963240 26815411

[132] Sharma M Boytard L Hadi T Koelwyn G Simon R Ouimet M. Enhanced glycolysis and HIF-1α activation in adipose tissue macrophages sustains local and systemic interleukin-1β production in obesity. Sci Rep. (2020) 10:5555. doi: 10.1038/s41598-020-62272-9 32221369 PMC7101445

[133] Hildebrandt X Ibrahim M Peltzer N. Cell death and inflammation during obesity: "Know my methods, WAT(son)". Cell Death Differ. (2023) 30:279–92. doi: 10.1038/s41418-022-01062-4 PMC9520110 36175539

[134] Qiao YC Chen YL Pan YH Ling W Tian F Zhang XX. Changes of transforming growth factor beta 1 in patients with type 2 diabetes and diabetic nephropathy: A PRISMA-compliant systematic review and meta-analysis. Med (Baltimore). (2017) 96:e6583. doi: 10.1097/MD.0000000000006583 PMC5403085 28403088

[135] Whitehead GS Thomas SY Nakano K Royer DJ Burke CG Nakano H. A neutrophil/TGF-β axis limits the pathogenicity of allergen-specific CD4+ T cells. JCI Insight. (2022) 7:e150251. doi: 10.1172/jci.insight.150251 35191395 PMC8876454

[136] Fain JN Tichansky DS Madan AK. Transforming growth factor beta1 release by human adipose tissue is enhanced in obesity. Metabolism. (2005) 54:1546–51. doi: 10.1016/j.metabol.2005.05.024 16253647

[137] Riester M Xu Q Moreira A Zheng J Michor F Downey RJ. The Warburg effect: persistence of stem-cell metabolism in cancers as a failure of differentiation. Ann Oncol. (2018) 29:264–70. doi: 10.1093/annonc/mdx645 PMC6658717 29045536

[138] Sancho P Barneda D Heeschen C. Hallmarks of cancer stem cell metabolism. Br J Cancer. (2016) 114:1305–12. doi: 10.1038/bjc.2016.152 PMC4984474 27219018

[139] Yasuda T Ishimoto T Baba H. Conflicting metabolic alterations in cancer stem cells and regulation by the stromal niche. Regener Ther. (2021) 17:8–12. doi: 10.1016/j.reth.2021.01.005 PMC7851492 33598509

[140] Fredriksson K Fielhaber JA Lam JK Yao X Meyer KS Keeran KJ. Paradoxical effects of rapamycin on experimental house dust mite-induced asthma. PloS One. (2012) 7:e33984. doi: 10.1371/journal.pone.0033984 22685525 PMC3368343

[141] Wu Y Wang W Peng XM He Y Xiong YX Liang HF. Rapamycin upregulates connective tissue growth factor expression in hepatic progenitor cells through TGF-β-smad2 dependent signaling. Front Pharmacol. (2018) 9:877. doi: 10.3389/fphar.2018.00877 30135653 PMC6092675

[142] van der Poel HG. Mammalian target of rapamycin and 3-phosphatidylinositol 3-kinase pathway inhibition enhances growth inhibition of transforming growth factor-beta1 in prostate cancer cells. J Urol. (2004) 172:1333–7. doi: 10.1097/01.ju.0000138829.97838.19 15371835

[143] Osman B Doller A Akool e-S Holdener M Hintermann E Pfeilschifter J. Rapamycin induces the TGFbeta1/Smad signaling cascade in renal mesangial cells upstream of mTOR. Cell Signal. (2009) 21:1806–17. doi: 10.1016/j.cellsig.2009.07.016 19666112

[144] Steagall WK Stylianou M Pacheco-Rodriguez G Yu ZX Moss J. Unexpected sirolimus-stimulated airway hyperreactivity in lymphangioleiomyomatosis. ERJ Open Res. (2023) 9:00305–2023. doi: 10.1183/23120541.00305-2023 PMC10423980 37589458

[145] Hansen G McIntire JJ Yeung VP Berry G Thorbecke GJ Chen L. CD4(+) T helper cells engineered to produce latent TGF-beta1 reverse allergen-induced airway hyperreactivity and inflammation. J Clin Invest. (2000) 105:61–70. doi: 10.1172/JCI7589 10619862 PMC382583

[146] Nemeth K Keane-Myers A Brown JM Metcalfe DD Gorham JD Bundoc VG. Bone marrow stromal cells use TGF-beta to suppress allergic responses in a mouse model of ragweed-induced asthma. Proc Natl Acad Sci U S A. (2010) 107:5652–7. Gorham, Jared D [corrected to Gorham, James D]; Bundoc, Victor G [corrected to Bundoc, Virgilio G]. doi: 10.1073/pnas.0910720107 PMC2851758 20231466

[147] Rangarajan S Kurundkar A Kurundkar D Bernard K Sanders YY Ding Q. Novel mechanisms for the antifibrotic action of nintedanib. Am J Respir Cell Mol Biol. (2016) 54:51–9. doi: 10.1165/rcmb.2014-0445OC PMC4742925 26072676

[148] Lee HY Hur J Kim IK Kang JY Yoon HK Lee SY. Effect of nintedanib on airway inflammation and remodeling in a murine chronic asthma model. Exp Lung Res. (2017) 43:187–96. doi: 10.1080/01902148.2017.1339141 28696800

[149] Wei Y Zhang Z Wang F Zhou S. Assessment of tumor growth factor-β1 neutralizing antibody in the treatment of allergic rhinitis and asthma. Exp Ther Med. (2018) 15:649–56. doi: 10.3892/etm.2017.5501 PMC5772626 29399067

[150] Hamidi SH Kadamboor Veethil S Hamidi SH. Role of pirfenidone in TGF-β pathways and other inflammatory pathways in acute respiratory syndrome coronavirus 2 (SARS-Cov-2) infection: a theoretical perspective. Pharmacol Rep. (2021) 73:712–27. doi: 10.1007/s43440-021-00255-x PMC8057922 33880743

[151] Mansoor JK Decile KC Giri SN Pinkerton KE Walby WF Bratt JM. Influence of pirfenidone on airway hyperresponsiveness and inflammation in a Brown-Norway rat model of asthma. Pulm Pharmacol Ther. (2007) 20:660–8. doi: 10.1016/j.pupt.2006.07.005 17049446

[152] Halwani R Sultana A Vazquez-Tello A Jamhawi A Al-Masri AA Al-Muhsen S. Th-17 regulatory cytokines IL-21, IL-23, and IL-6 enhance neutrophil production of IL-17 cytokines during asthma. J Asthma. (2017) 54:893–904. doi: 10.1080/02770903.2017.1283696 28635548

[153] Wang J Zhao X Wan YY. Intricacies of TGF-β signaling in Treg and Th17 cell biology. Cell Mol Immunol. (2023) 20:1002–22. doi: 10.1038/s41423-023-01036-7 PMC10468540 37217798

[154] Margelidon-Cozzolino V Tsicopoulos A Chenivesse C de Nadai P. Role of th17 cytokines in airway remodeling in asthma and therapy perspectives. Front Allergy. (2022) 3:806391. doi: 10.3389/falgy.2022.806391 35386663 PMC8974749

[155] Lajoie S Lewkowich I Herman NS Sproles A Pesce JT Wynn TA. IL-21 receptor signalling partially mediates Th2-mediated allergic airway responses. Clin Exp Allergy. (2014) 44:976–85. doi: 10.1111/cea.12341 PMC4083497 24807637

[156] Alcorn JF Rinaldi LM Jaffe EF van Loon M Bates JH Janssen-Heininger YM. Transforming growth factor-beta1 suppresses airway hyperresponsiveness in allergic airway disease. Am J Respir Crit Care Med. (2007) 176:974–82. doi: 10.1164/rccm.200702-334OC PMC2078678 17761617

[157] McMillan SJ Xanthou G Lloyd CM. Manipulation of allergen-induced airway remodeling by treatment with anti-TGF-beta antibody: effect on the Smad signaling pathway. J Immunol. (2005) 174:5774–80. doi: 10.4049/jimmunol.174.9.5774 15843580

[158] Gomez G Ramirez CD Rivera J Patel M Norozian F Wright HV. TGF-beta 1 inhibits mast cell Fc epsilon RI expression. J Immunol. (2005) 174:5987–93. doi: 10.4049/jimmunol.174.10.5987 PMC1391973 15879091

[159] Fabregat I Moreno-Càceres J Sánchez A Dooley S Dewidar B Giannelli G. IT-LIVER Consortium. TGF-β signalling and liver disease. FEBS J. (2016) 283:2219–32. doi: 10.1111/febs.13665 26807763

[160] Chaker AM Zissler UM Poulos N Wagenmann M Bas M Gürth F. Activin-A is a pro-inflammatory regulator in type-2-driven upper airway disease. Int Arch Allergy Immunol. (2018) 176:15–25. doi: 10.1159/000487930 29656291

[161] Zhang F Qi Y Li J Liu B Liu Z Cui X. Activin A induces apoptosis of human lung adenocarcinoma A549 cells through endoplasmic reticulum stress pathway. Oncol Rep. (2024) 51:29. doi: 10.3892/or.2023.8688 38131250 PMC10777458

[162] Compalati E Ridolo E Passalacqua G Braido F Villa E Canonica GW. The link between allergic rhinitis and asthma: the united airways disease. Expert Rev Clin Immunol. (2010) 6:413–23. doi: 10.1586/eci.10.15 20441427

[163] Togias A. Unique mechanistic features of allergic rhinitis. J Allergy Clin Immunol. (2000) 105:S599–604. doi: 10.1067/mai.2000.106885 10856164

[164] Neveu WA Allard JL Raymond DM Bourassa LM Burns SM Bunn JY. Elevation of IL-6 in the allergic asthmatic airway is independent of inflammation but associates with loss of central airway function. Respir Res. (2010) 11:28. doi: 10.1186/1465-9921-11-28 20205953 PMC2842243

[165] Woo J Koziol-White C Panettieri R Jr Jude J. TGF-β: The missing link in obesity-associated airway diseases? Curr Res Pharmacol Drug Discovery. (2021) 2:100016. doi: 10.1016/j.crphar.2021.100016 PMC8663968 34909651

